# Characterizing Head Acceleration Events in Law Enforcement Cadets During Subject Control Technique Training

**DOI:** 10.1007/s10439-023-03382-z

**Published:** 2023-10-17

**Authors:** Carly R. Smith, James A. Onate, Nathan A. Edwards, Joshua A. Hagen, Chris Kolba, Scott Paur, Joshua Walters, Jaclyn B. Caccese

**Affiliations:** 1 School of Health and Rehabilitation Sciences, The Ohio State University, Columbus, OH USA; 2 Human Performance Collaborative, The Ohio State University, Columbus, OH USA; 3 Chronic Brain Injury Program, The Ohio State University, Columbus, OH USA; 4 Wexner Medical Center, The Ohio State University, Columbus, OH USA; 5 Franklin County Sheriff’s Office, Columbus, OH USA

**Keywords:** Repetitive head impacts, Concussion, Police, Combat training

## Abstract

Law enforcement cadets (LECs) complete weeks of subject control technique training. Similar sport-related combat training has been shown to expose participants to head acceleration events (HAEs) that have potential to result in short- and long-term impairments. The purpose of this study was to describe the number and magnitude of HAEs in LECs throughout their training. 37 LECs (7 females; age = 30.6 ± 8.8 years; BMI = 30.0 ± 6.0) were recruited from a law enforcement organization. Participants wore instrumented mouthguards, which recorded all HAEs exceeding a resultant 5 g threshold for training sessions with the potential for HAEs. Participants completed three defensive tactics (DT) training sessions, a DT skill assessment (DTA), and three boxing sessions. Outcome measures included the number of HAEs, peak linear acceleration (PLA), and peak rotational velocity (PRV). There were 2758 true-positive HAEs recorded across the duration of the study. Boxing sessions accounted for 63.7% of all true-positive HAEs, while DT accounted for 31.4% and DTA accounted for 4.9%. Boxing sessions resulted in a higher number of HAEs per session (*F*_2,28_ = 48.588, *p* < 0.001, *η*_p_^2^ = 0.776), and higher median PLA (*F*_2,28_ = 8.609, *p* = 0.001, η_p_^2^ = 0.381) and median PRV (*F*_2,28_ = 11.297, *p* < 0.001, *η*_p_^2^ = 0.447) than DT and DTA. The LECs experience a high number of HAEs, particularly during boxing sessions. Although this training is necessary for job duties, HAE monitoring may lead to modifications in training structure to improve participant safety and enhance recovery.

## Introduction

During the training academy, law enforcement cadets (LECs) complete weeks of subject control technique training, including defensive tactics (DT) and boxing. Subject control techniques are an important aspect of training for cadets. These techniques are designed to test the cadet’s critical thinking, train acute stress management, and teach proper use of force under pressure. However, subject control technique training also exposes cadets to repetitive head impacts (RHIs) and has the potential to result in concussions/mild traumatic brain injuries (mTBIs). These types of training drills cannot be eliminated since having defensive skills is a critical component of law enforcement job performance. Nonetheless, characterizing RHI exposure during training may provide ways to mitigate injury, ultimately protecting law enforcement officer’s operational capability and career longevity [1]. Head acceleration measurement may also help training academy leaders ensure that cadets are using proper technique and not putting themselves or others at risk of injury during this critical training.

A growing body of literature quantifies RHI exposure using wearable devices containing accelerometers to measure linear acceleration and gyroscopes to measure rotational velocity. Almost exclusively, these studies quantify head acceleration events (HAEs) within the sports population (e.g., in football and soccer athletes) [2–8] using head-mounted sensors [6], helmet-affixed sensors [2, 5], and instrumented mouthguards [3, 4, 8]. In contrast to sport-related concussions (SRC), these sport-related RHIs, sometimes referred to as subconcussive head impacts, oftentimes do not result in clinical manifestations [9, 10]. However, there is growing evidence that sustaining RHIs have the potential to result in short- and long-term neuropsychological and neurophysiological impairments, including behavioral changes, altered cognitive performance, and alterations to neural structure and function [11–13].

Although researchers have previously described HAEs in sport, HAEs in other settings (e.g., military and civilian tactical training) remain understudied [14, 15]. Sport-related combat (e.g., boxing and mixed martial arts) has been shown to expose participants to 6-19 HAEs per exposure and magnitudes of up to approximately 40 g median peak linear acceleration and 3700 rad/s^2^ median peak angular acceleration [11, 16]. Therefore, the purpose of this study was to describe the number and magnitude of HAEs in civilian LECs during the training academy. We hypothesized that there would be higher magnitude HAEs during boxing because of the nature of the training, but a higher total number of HAEs in DT training because of longer duration training sessions. Overall, head acceleration measurement is an important tool for protecting the health, safety, and well-being of LECs during the training academy.

## Methods

### Participants

A total of 40 LECs from one training class (fall 2022) were invited to participate. Two LECs were excluded from participation because they wore dental braces and could not participate in the boil-and-bite process for mouthguard fitting. There were no other exclusionary criteria to maximize generalizability. One LEC sustained an injury during training and could not continue in the training academy; therefore, his data were removed from analyses. There were 37 LECs [7 females, 30 males; age = 30.6 ± 8.8 years; weight = 94.8 ± 24.9 kilograms; height = 176.8 ± 9.1 centimeters; 78.4% White, non-Hispanic (29/37), 10.8% Black (4/37), 5.4% Hispanic (2/37), and 5.4% other races not described above (2/37)] enrolled in the study and included in analyses. Prior to participation, all participants signed an informed consent form that was approved by the University’s Institutional Review Board (Study ID 2022H0299). Data collection procedures are reported in accordance with the Consensus Head Acceleration Measurement Practices 2022 checklist [17].

### Instrumentation

This study used the Impact Monitoring Mouthguard (V1; Prevent Biometrics, Edina, MN), which measured peak linear acceleration (PLA) up to 200 g via a tri-axial accelerometer at 3200 Hz and peak rotational velocity (PRV) up to 35 rad/s via a tri-axial gyroscope at 3200 Hz. The instrumented mouthguards (IMGs) had a trigger threshold of a resultant 5 g impact. When a HAE above this threshold occurred, data were recorded from 10 ms of pre-trigger to 40 ms of post-trigger. Any HAEs below the threshold were not recorded. Following data collection, processed data were downloaded with a 5 g inclusion threshold and used in data analyses. Although a 10 g threshold is more common in sport literature, a 5 g threshold was utilized in this study because data collections occurred in a more controlled environment than is typical in sports and LECs wore IMGs only when they were involved in training drills with potential for exposure to HAEs. Therefore, the potential for false-positive HAEs was lower and a 5 g threshold ensured that all HAEs were recorded [19–23].

Each participant was fitted for an IMG through a standard boil-and-bite process by trained research team members. All IMGs were assessed to ensure a proper fit, meaning they fit snugly on the participants’ upper teeth and were not displaced when the participants opened their mouth. Independent validation suggests that PLA (*r*^2^ = 0.99) and PRV (*r*^2^ = 0.92) measurements recorded from the Impact Monitoring Mouthguards were highly correlated with an anthropomorphic test device at 25, 50, 75, and 100 g [18]. The mean relative error in peak magnitude was 2.5% for PLA and 4.6% for PRV, which is better than other commercially available systems for measuring HAEs [18]. Data were processed according to Prevent Biometrics’ custom algorithm (file format version 2.2), which assigned a quality flag (i.e., 0, 1, 2) to each HAE based on movement of the IMG relative to the teeth during the HAE and then applied a low-pass filter accordingly (i.e., 0 = 200 Hz, 1 = 100 Hz, 2 = 50 Hz filter).

### Data Collection Sessions

**Fig. 1:**
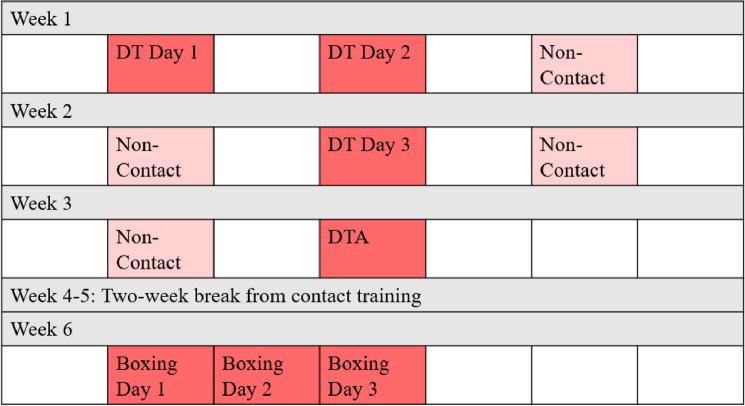
Training schedule, including defensive tactics (DT), defensive tactics assessment (DTA), and boxing. Contact and non-contact sessions are differentiated by color (i.e., darker color, contact session; lighter color, non-contact session)

Participants completed DT training three times per week for three weeks including a final DT session as a skill assessment (DTA). Of the seven total DT sessions, only three training sessions and the DTA had the potential for RHIs. Following a two-week break from DT training, participants then completed three consecutive days of boxing training. All three days of boxing had the potential for RHIs. Therefore, participants wore IMGs for a total of seven sessions with the potential for RHIs (Fig. 1). The DT training sessions included instruction and practice of subject control techniques (e.g., grappling), with each session being four hours in total length with varying duration of contact training. The DTA included two 2-min rounds in which cadets attempted to gain control over a simulated assailant using the DT skills learned in training. Some DTAs were abbreviated because subject control was gained quickly. During boxing sessions, participants only boxed against opponents for three, 1-min rounds each day. During these rounds, participants wore standard 16 oz boxing gloves and head gear, in addition to IMGs. Prior to each session, a member of the research team verified that each IMG was fully charged and functional. The number of participants for each test date varied due to participant injuries and unexpected absences, LEC removal from the training academy (from DTA failure), and equipment malfunction (i.e., battery failure, sensor breakage).

### Event Verification

Although video verification is considered best practice for HAE measurement [17], we were unable to video record sessions due to the sensitive nature of this training. Participants wore IMGs only during contact training with the potential for RHIs and removed IMGs during instructional and rest times. Furthermore, a member of the research team was present for each training session and took time-stamped notes. Additionally, prior research in combat sports has shown that once inactive-time recorded HAEs were removed, the number of false-positive reported were significantly reduced [23]. The research team also viewed HAEs in real time and noted false-positive HAEs. Prevent Biometrics’ proprietary black-box machine learning algorithm was also used to remove false-positive HAEs.

## Statistical Analysis

Outcome measures included number of HAEs, PLA, and PRV. Primary descriptive analyses report data relative to a 5 g threshold by date (DT 1, DT 2, DT 3, DTA, boxing 1, boxing 2, boxing 3) and session type (DT, three training days; DTA, single assessment day; boxing, three days of boxing) (Table 1). Data relative to a 10 g and 15 g threshold are also provided (Table 2) to allow for comparisons with prior research. [11, 24]

**Table 1:** Head acceleration events by date and session type at a 5 g threshold

Head impact number and magnitude by date and session type (5 g threshold)											
	True-positive impacts (*N*)	Participants (*N*)	Number of impacts per participant			Peak linear acceleration (PLA, g)			Peak rotational velocity (PRV, rad/s)		
			Median (IQR)	Minimum	Maximum	Median (IQR)	Minimum	Maximum	Median (IQR)	Minimum	Maximum
Date											
DT 1	434	37	11 (4–8)	0	37	12.5 (8.5–19.0)	5.0	61.0	7.8 (4.7–11.7)	0.8	30.6
DT 2	341	37	8 (2–11)	0	39	8.0 (6.4–11.2)	5.0	53.4	4.0 (2.6–6.6)	0.3	22.3
DT 3	92	30	3 (0–5)	0	13	9.4 (6.8–12.4)	5.2	25.8	6.4 (3.5–9.4)	0.6	15.6
DTA	134	32	2 (1–5)	0	19	10.8 (8.2–15.3)	5.3	55.0	4.3 (2.7–6.5)	1.0	45.7
Boxing 1	430	35	10 (6–18)	0	37	11.4 (8.4–16.5)	5.0	54.6	6.9 (4.4–10.1)	0.7	33.2
Boxing 2	598	33	17 (8–27)	0	59	12.4 (8.8–19.2)	5.0	70.6	8.0 (5.3–11.5)	0.4	39.9
Boxing 3	729	29	23 (18–29)	10	49	14.2 (9.9–21.1)	5.0	91.7	8.8 (5.8–12.2)	0.5	49.1
Session											
DT	867		22 (10–30)	0	72	10.1 (7.1–14.6)	5.0	61.0	6.1 (3.4–9.5)	0.3	30.6
DTA	134		2 (1–5)	0	19	10.8 (8.2–15.3)	5.3	55.0	4.3 (2.7–6.5)	1.0	45.7
Boxing	1757		48 (39–65)	2	124	12.9 (9.0–19.2)	5.0	91.7	8.0 (5.3–11.5)	0.4	49.1
Overall	2758		67 (42–102)	2	193	11.7 (8.3–17.7)	5.0	91.7	7.3 (4.4–1.08)	0.3	49.1

Values presented as median (interquartile range)

*DT* defensive tactics, *DTA* defensive tactics assessment, *PLA* peak linear acceleration, *PRV* peak rotational velocity.

**Table 2:** Head acceleration event descriptives by date and session type at 10 g and 15 g threshold

Head impact number and magnitude by date and session type (10 g threshold)											
	True-positive impacts (*N*)	Participants (*N*)	Number of impacts per participant			Peak linear acceleration (PLA, g)			Peak rotational velocity (PRV, rad/s)		
			Median (IQR)	Minimum	Maximum	Median (IQR)	Minimum	Maximum	Median (IQR)	Minimum	Maximum
Date											
DT 1	283	37	6 (2–11)	0	22	16.6 (12.7–23.4)	10.0	61.0	9.3 (6.3–13.4)	1.5	30.6
DT 2	115	37	3 (1–5)	0	9	13.1 (11.1–16.0)	10.0	53.4	5.8 (4.0–9.1)	2.1	20.3
DT 3	42	30	1 (0–2)	0	6	12.6 (11.2–14.6)	10.3	25.8	8.8 (5.0–11.2)	2.6	15.6
DTA	76	32	1 (0–4)	0	14	14.3 (11.8–18.5)	10.0	55.0	5.1 (3.2–7.7)	1.0	45.7
Boxing 1	267	35	6 (3–14)	0	23	15.1 (12.0–20.2)	10.0	54.6	8.0 (5.9–11.4)	1.2	33.2
Boxing 2	396	33	9 (5–18)	0	38	16.8 (12.5–22.2)	10.0	70.6	9.5 (6.7–13.4)	1.9	39.9
Boxing 3	541	29	17 (14–23)	7	34	16.9 12.8–23.9)	10.0	91.7	9.5 (6.7–13.3)	0.9	49.1
Session											
DT	440		10 (5–18)	0	36	14.6 (12.0–20.4)	10.0	61.0	8.4 (5.3–12.0)	1.5	30.6
DTA	76		1 (0–4)	0	14	14.3 (11.8–18.5)	10.0	55.0	5.1 (3.2–7.7)	1.0	45.7
Boxing	1204		27 (19–36)	0	68	16.5 (12.5–22.6)	10.0	91.7	9.1 (6.4–13.0)	0.9	49.1
Overall	1720		42 (31–64)	1	113	15.9 (12.3–21.9)	10.0	91.7	8.8 (5.9–12.6)	0.9	49.1

Head impact number and magnitude by date and session type (15 g threshold)											
	True-positive impacts (*N*)	Participants (*N*)	Number of impacts per participant			Peak linear acceleration (PLA, g)			Peak rotational velocity (PRV, rad/s)		
			Median (IQR)	Minimum	Maximum	Median (IQR)	Minimum	Maximum	Median (IQR)	Minimum	Maximum
Date											
DT 1	167	37	4 (1–7)	0	13	21.7 (17.5–26.9)	15.0	61.0	10.9 (7.6–14.9)	2.6	30.6
DT 2	34	37	0 (0–2)	0	5	18.9 (17.0–22.8)	15.3	53.4	7.9 (4.7–11.2)	2.3	20.3
DT 3	9	30	0 (0–0)	0	2	16.8 (16.6–19.7)	15.4	25.8	11.1 (10.1–11.9)	3.6	13.9
DTA	35	32	0 (0–2)	0	7	18.9 (17.1–22.0)	15.1	55.0	5.8 (4.3–10.1)	1.0	45.7
Boxing 1	137	35	3 (0–7)	0	14	20.0 (17.1–24.0)	15.0	54.6	10.1 (7.0–14.2)	2.2	33.2
Boxing 2	235	33	5 (2–11)	0	25	20.8 (17.7–27.5)	15.0	70.6	10.9 (7.7–14.9)	1.9	39.9
Boxing 3	340	29	11 (9–14)	2	27	22.1 (17.3–29.1)	15.0	91.7	11.1 (8.1–15.6)	0.9	49.1
Session											
DT	210		4 (1–9)	0	18	21.0 (17.1–25.7)	15.0	61.0	10.6 (7.2–14.0)	2.3	30.6
DTA	35		0 (0–2)	0	7	18.9 (17.1–22.0)	15.1	55.0	5.8 (4.3–10.1)	1.0	45.7
Boxing	712		19 (13–28)	0	62	21.1 (17.5–26.6)	15.0	91.7	10.8 (7.7–15.0)	0.9	49.1
Overall	957		27 (14–33)	0	67	20.8 (17.4–26.4)	15.0	91.7	10.7 (7.4–14.5)	0.9	49.1

Values presented as median (interquartile range)

*DT* defensive tactics, *DTA* defensive tactics assessment, *PLA* peak linear acceleration, *PRV* peak rotational velocity

To compare HAEs across session types, we used a repeated-measures analysis of variance (rmANOVA). Dependent variables were the number of HAEs per session, median PLA (mPLA), and median PRV (mPRV). The independent variable was session type: DT, DTA, and boxing. Significance was defined a priori *p* < 0.05. We used a Bonferroni correction for multiple comparisons (i.e., number of HAEs, PLA, PRV); therefore, corrected significance was 0.05/3 = 0.017. For each comparison, we also provide effect sizes as partial eta squared with 0.01 considered a small effect, 0.06 considered a medium effect, and 0.14 considered a large effect. A total of 7 participants were excluded from the rmANOVA due to the participants being removed from the training academy (*n* = 2), or not participating in all sessions or IMG technical error resulting in no recorded HAEs (*n* = 5). Statistical analyses were completed using SPSS statistical software (IBM SPSS Statistics for Windows, version 28.0.1.1. Armonk, NY: IBM Corp).

## Results

**Fig. 2:**
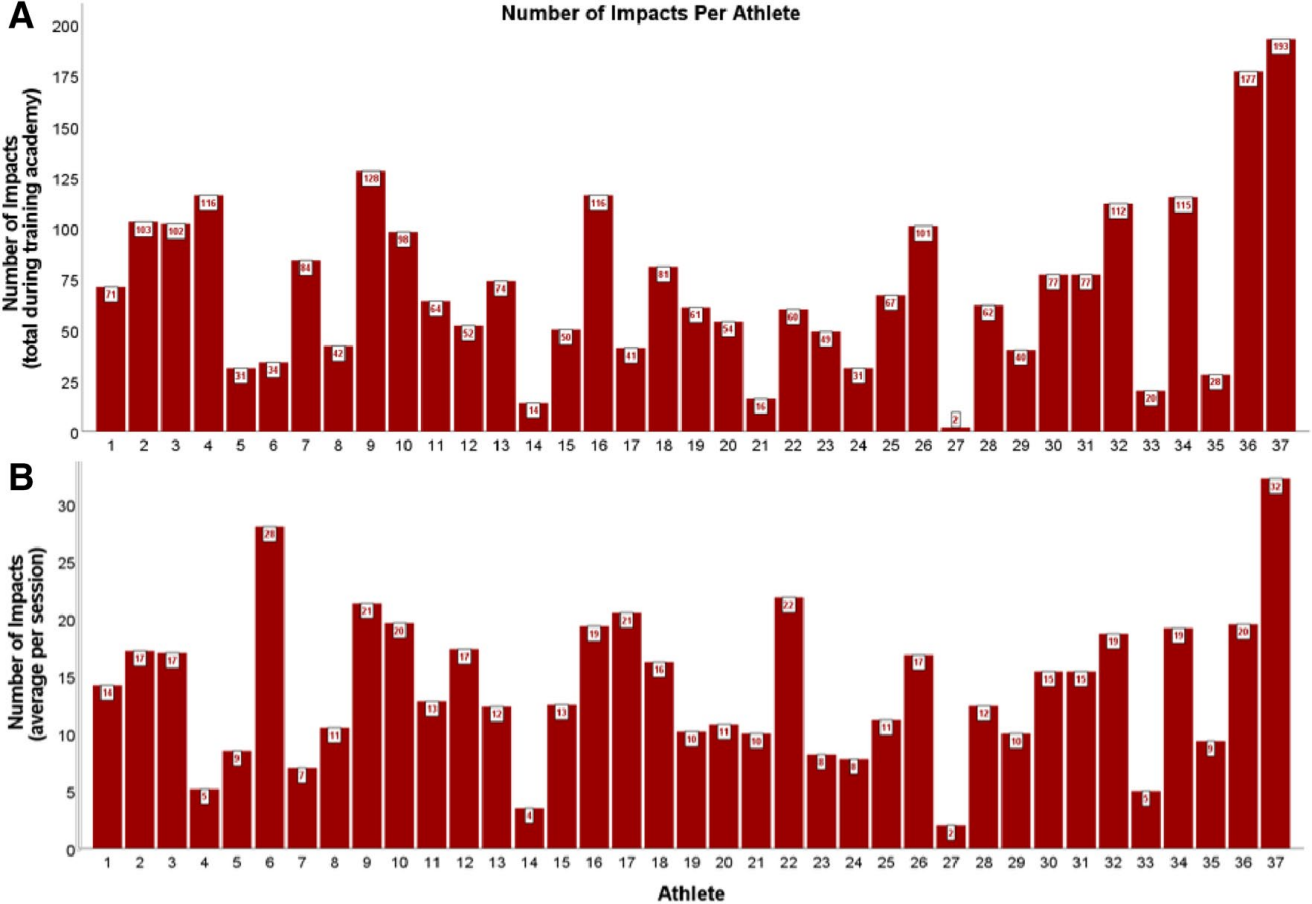
**a** Total number of impacts per athlete across the duration of training academy. **b** Average number of impacts per athlete per session during the training academy

**Fig. 3:**
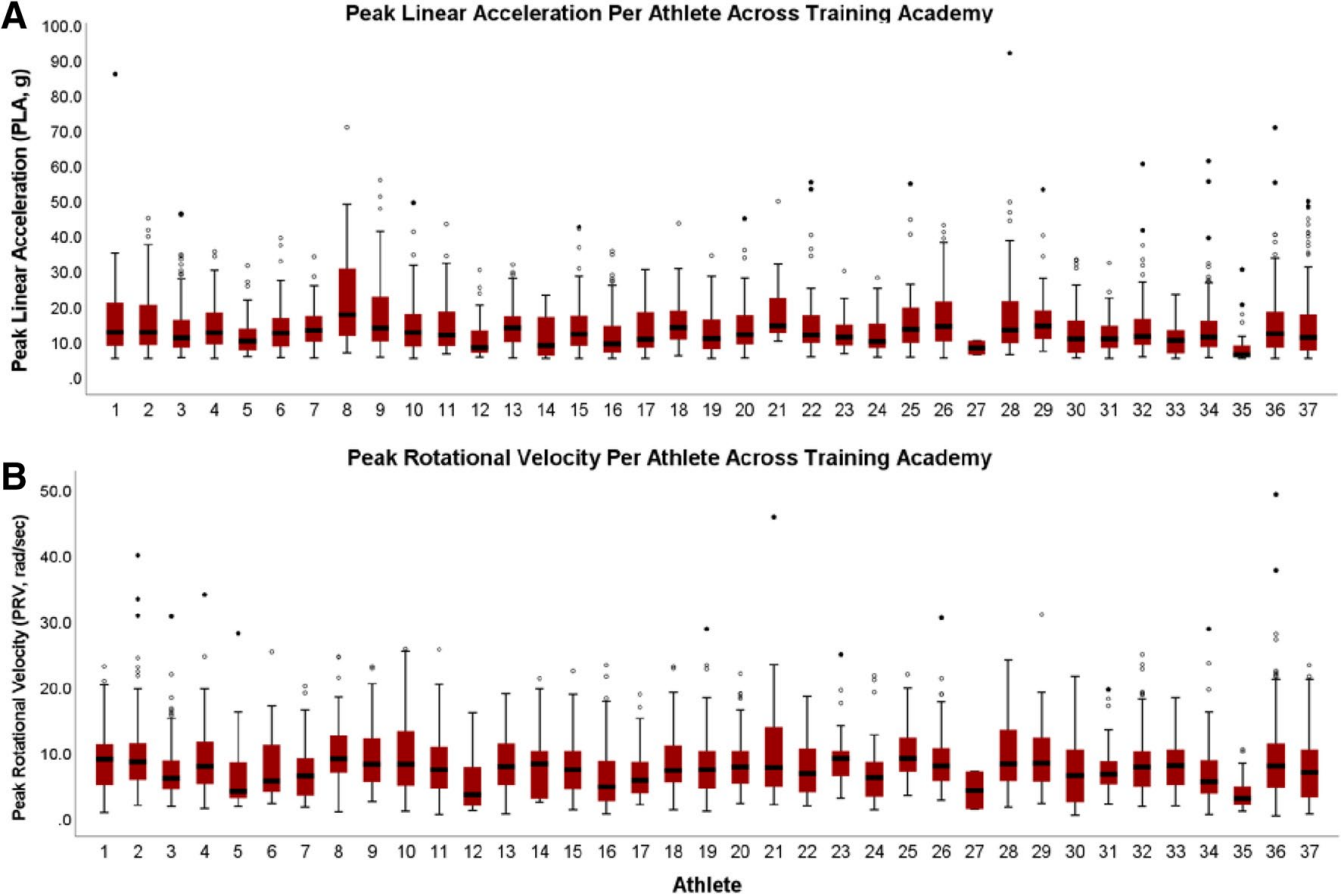
**a** Peak linear acceleration (g) for all participants across the training academy. **b** Peak rotational velocity (rad/s) for all participants across the training academy

Table 1 provides descriptive statistics of number and magnitude (i.e., PLA in g; PRV in rad/s) for HAEs > 5 g threshold deemed to be true-positives by date and session type. Descriptive statistics for 10 g and 15 g thresholds are presented in Table 2. There were a total of 5761 HAEs recorded > 5 g threshold across the duration of the study; 2758 (48.9%) HAEs were deemed to be true-positives. Boxing sessions accounted for 63.7% (1757/2758) of all true-positive HAEs. In comparison, DT and DTA accounted for 31.4% and 4.9% of HAEs, respectively. Boxing resulted in a significantly higher number of HAEs per session (*F*_2,28_ = 48.588, *p* < 0.001, *η*_p_^2^ = 0.776), mPLA (*F*_2,28_ = 8.609, *p* = 0.001, *η*_p_^2^ = 0.381), and mPRV (*F*_2,28_ = 11.297, *p* < 0.001, *η*_p_^2^ = 0.447) than DT and DTA. Figures 2 and 3 present number and magnitude (i.e., PLA in g; PRV in rad/s), respectively, for all HAEs deemed to be true-positives on an individual participant basis across all training academy sessions.

## Discussion

The purpose of this study was to describe the number and magnitude of HAEs sustained during the training academy in LECs. We hypothesized that there would be higher magnitude HAEs during boxing, but a higher number of HAEs during DT training. Our hypotheses were partially confirmed in that both the number and magnitude of HAEs was higher in boxing sessions. It is important to note that this is the first study to quantify HAEs in law enforcement cadets during the training academy. Findings provide valuable insights into HAEs in occupational settings for tactical athletes.

The HAEs sustained during boxing were significantly higher in number, mPLA, and mPRV compared to those sustained during both DT and DTA. These differences are likely attributed to the type of training; one of the aims of boxing involves striking the opponent. Comparatively, the aim of both DT and DTA was achieving subject control, where impacts to the head were incidental rather than intentional. Although there is not a robust body of literature on this topic, one prior study [11] compared boxing to mixed martial arts (MMA), which combines jujitsu, wrestling, boxing, and other martial arts. The prior study reported 14 HAEs per athlete per boxing session for three, 1-min rounds with a threshold of 15 g, while the current study demonstrated approximately 17 HAEs per LEC during boxing sessions in the same time frame with a 5 g threshold (approximately 7 HAEs with a 15 g threshold, see Tables 1 and 2). Similarly, the prior study reported a mPLA of 19.3 g and mPRA of 1596 rad/s^2^ for boxing for MMA with a threshold of 15 g, while the current study demonstrated a mPLA of 12.9 g and mPRV of 8.0 rad/s for boxing, with a 5 g threshold (approximately a mPLA of 21 g and a mPRV of 11 rad/s with a 15 g threshold, see Tables 1 and 2). Of note, the prior study presented mPRA, whereas the present study presented mPRV. Nonetheless, taken together, findings suggest that the number of HAEs during boxing training in LECs is approximately half that of sport boxing, though the magnitude of HAEs during boxing was comparable to sport boxing. Thus, further exploration of HAEs in boxing is warranted for both LECs and sports populations to ensure appropriate consideration of participant safety.

Although the skills taught during the training academy are necessary for this occupation, this study highlights the importance of training schedule structures. Boxing resulted in the highest number and magnitude of HAEs, and yet the time between training sessions was the shortest. Specifically, there was at least 24 hours between DT and DTA sessions for recovery, while boxing training occurred on three consecutive days. Head acceleration measurement in football suggests three possible mechanisms associated with sport-related concussions: (1) a single, high magnitude impact [25–27], with differences in individual tolerance or personalized concussion thresholds [28]; (2) a higher number of HAEs on the day of the diagnosed concussion [5]; and (3) a shorter time between HAEs on the day of the diagnosed concussion [29]. Furthermore, studies have reported a cumulative effect of HAEs, including long-term consequences even without concussion occurrence [9, 29]. Therefore, modifying training schedules to allow for more recovery between sessions, particularly between boxing sessions, presents an opportunity to reduce the cumulative effect of HAEs and the risk of concussion.

**Fig. 4:**
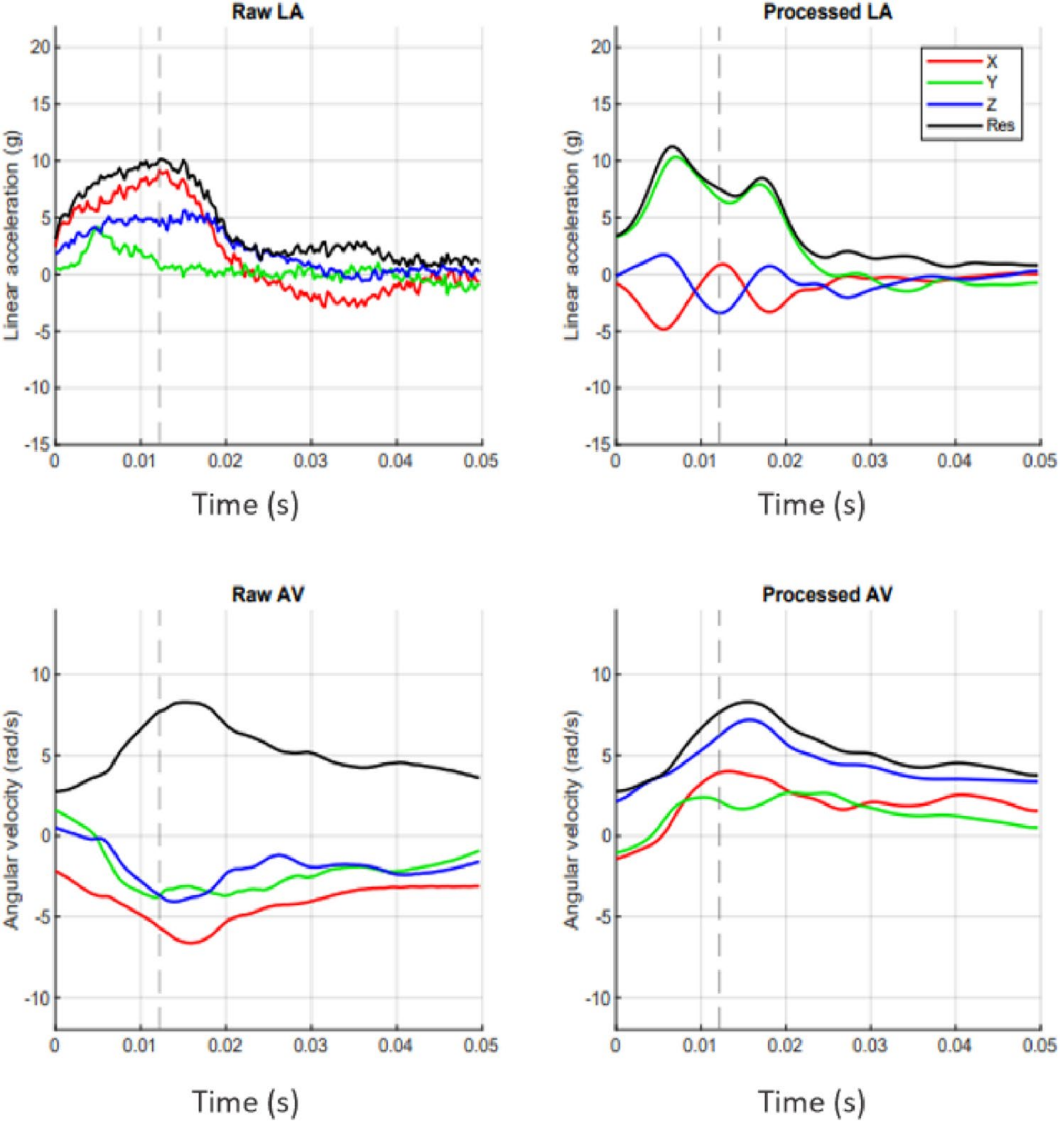
Example head acceleration event waveform, 11.3 g lasting 20 ms. Raw (unprocessed) linear acceleration (LA) and angular velocity (AV) data are presented on the left. Processed LA and AV, on the right, were aligned to the head’s coordinate system, transformed the center of gravity to the head, filtered with a cutoff frequency of 200 Hz, and according to any other processing done by Prevent’s proprietary algorithm. Traces include the X (red), Y (green), Z (blue), and resultant (black) waveforms

These data (specifically the maximum PLA recorded) also support the need for on-site medical providers during these training sessions. The maximum PLA recorded was 91.7 g and occurred during a boxing session. Although there is no established threshold for sustaining a concussion, a meta-analysis reported that the mean PLA associated with a football-related concussion was 98.7 g and the mean PRA was 5776.6 rad/s^2^ [25, 30–33]. It is important to note that prior research suggests the duration of HAEs in combat sports is longer (e.g., 20 ms, Fig. 4) than HAEs in football (e.g., 15 ms), so the PLA and PRA resulting in concussion may be lower [34]. Further, there is likely an individual threshold for concussion [28, 35]. Regardless of where an individual’s threshold may truly lie, the HAEs recorded during boxing in the training academy are within the range where concussion is possible. Though there were no diagnosed concussions during this study, concussions may be under-reported due to the nature of this training being that not completing training does not allow them to progress to the next stage of their career, as well as the fact that there were no medical providers on-site for assessment. The ramifications of concussion without immediate removal and adequate follow-up care are well established, including prolonged recovery times and more severe symptoms [36–41]. Therefore, having a medical provider on-site for concussion diagnosis would increase the likelihood of LECs receiving appropriate concussion management, thereby providing an opportunity to decrease risk for long-term impairments.

On an individual cadet level, there was high variability in number of HAEs, mPLA, and mPRV across the duration of the training academy. Although not explored in this study, there is likely a benefit to determining underlying mechanisms causing this variability. Considerations of subject characteristics such as sex, size, and prior combat training may have implications on number and magnitude of HAEs. Prior work has explored HAEs between males and females during boxing and MMA sparring [11, 42]. These data were limited due to an unequal sample (few female participants due to the nature of the population), but preliminary analyses revealed female participants had a lower number but a similar magnitude of HAEs compared to their male counterparts. In non-combat sports, there is a lack of consensus if larger participant size results in higher or lower HAEs [43, 44], and the relationship between participant size and HAE number and magnitude has not been explored in combat sports. However, previous research has demonstrated that higher weight classes have higher injury rates [45]. One study proposed that greater experience in sport-specific participation had positive effects on shortening recovery times following concussions [46]. However, it remains unknown how previous experience or training affects HAE number and magnitude. Subject characteristics, both modifiable and non-modifiable listed above, may impact HAE quantity and magnitude and thus should be further investigated. Modifiable risk factors (i.e., weight/size, neck strength, etc.), specifically, may improve safety of those who are at higher risk of RHI exposure and concussion.

**Fig. 5:**
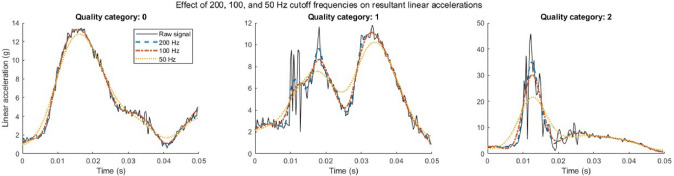
Three exemplar HAEs of high, moderate, and low quality filtered at 50 Hz, 100 Hz, and 200 Hz to examine the effect of each filtering frequency on linear acceleration (g)

This was the first study to examine HAEs in LECs during subject control technique training but was not without limitations. First was the inability to complete video verification of the HAEs leading to a reliance on the IMG manufacturer’s proprietary black-box filtering algorithms. Although video verification is considered a best practice [47], we used other methods (i.e., detailed, time-stamped notes and live monitoring of HAEs) to mitigate false-positive HAEs and ensure accurate reporting [23]. Additionally, we analyzed all recorded HAEs that occurred during three boxing days in a separate cohort of LECs and assigned true-positive and false-positive based upon time-stamped notes. This was done for only boxing because only 2 participants were active at one time; thus, we were able to identify active time of participation more accurately versus extraneous HAEs recorded outside of active time. We compared our findings to Prevent’s flagged true-positive HAEs. We found that at least 85% appeared to be correctly classified, 1% were classified incorrectly, and 14% were unable to be confirmed without video verification. Although video verification is the gold standard, this does assuage some concerns related to relying on Prevent’s classification algorithm for identifying HAEs. We also examined the effect of Prevent’s filtering algorithm. Specifically, Prevent assigns each HAE with a quality indicator: high (0), moderate (1), or low (2). This corresponds to the low-pass filter cutoff frequency used for each HAE: 200 Hz, 100 Hz, and 50 Hz, respectively, as well as an empirical correction factor of 1.0, 1.2, and 1.9 on the PLA (firmware version ‘2.0.17 scaled’). These cutoff frequencies were selected from frequency spectrum analyses in helmeted head impacts in which the frequency band of interest was 20-80 Hz [48]. Therefore, filtering at 200 Hz or 100 Hz should retain the signal energy for these types of impacts. Filtering at 50 Hz may be necessary for excessive noise and should retain most of the signal energy for the majority of helmeted impacts, but the correction factor is applied to better estimate the true peak. However, it is unclear how these cutoff frequencies and correction factors would affect non-helmeted impacts as quantified herein. To explore these effects, we exported the raw (unprocessed) PLA data and applied 50 Hz, 100 Hz, and 200 Hz zero-lag Butterworth filters to three exemplar HAEs (one of each quality, Fig. 5). There does not appear to be much effect of filtering a high-quality, “0,” impact at each cutoff frequency. There does appear to be a greater effect on filtering low-quality, “2,” impacts. However, we are unable to determine if this approach is removing only excessive, non-head impact-related noise on top of the signal of interest or if it is also artificially reducing the true magnitude of the HAE.

Second, this study used boil-and-bite instrumented mouthguards. Although these were used because they were more practical in this population, we acknowledge that custom-made mouthguards based on the individual’s dentition have been shown to improve the performance on-field applications. Validation of the boil-and-bite Prevent Biometrics Impact Monitor Mouthguard was done both in-laboratory and on-field. In-laboratory validation included a pendulum impactor to simulate helmeted and non-helmeted impacts to the head. In this prior work, a medium National Operating Committee on Standards for Athletic Equipment (NOCSAE) headform was attached to a Hybrid III 50th percentile male neck using a custom adapter and then mounted on a linear slide table with 5 degrees of freedom. Helmeted (rigid impactor) and non-helmeted (padded and rigid impactor) impacts occurred at the front, front boss, rear boss, and rear locations of the helmet or headform at target linear head accelerations of 25, 50, 75, and 100 g [18]. In validating sensors using anthropometric devices, compared to cadavers, there are simplifications that are made to mandibular anatomy that may affect the interaction between the mandible and IMG [49] that is not synonymous with actual human movement during impact. The boil-and-bite Prevent Biometrics Impact Monitor Mouthguard (CCC = 0.97) was also field-tested in helmeted-conditions with two collegiate football players across three practices with video recordings of practices [18]. When removing impacts recorded during non-active time, Prevent recorded 147 suspected impacts compared to 120 video verified impacts, resulting in a positive predictive value (PPV) of 81.6 % [18]. A potential explanation for this discrepancy between in-lab and on-field performance is the deterioration of the IMG because of prolonged, continuous use in sports such as rugby or football, in addition to structural damage caused by repeatedly biting the IMG, leading to reduced coupling between the IMG and skull. For this study, the concern for these negative effects is lessened as the participants wore IMGs only for seven days, with limited time of use across each day. This ranged from approximately 2–3, 1-min rounds daily to 15 min of total training time daily, based upon the training activities they were engaged in that day. Additionally, IMGs were visually inspected following each use to assess for signs of damage, potentially indicating poor fit, and replaced as needed to maintain optimal fit and function. However, we cannot fully exclude the hypothesis that the mouthguard’s material influences recorded head impact magnitudes.

Third was the limited number of female participants prohibiting sex comparisons. Future research in larger samples may be able to address sex as a biological variable. Fourth, all participants were in training; thus, we cannot generalize the findings to HAEs law enforcement officers may experience on-duty or during combat sports. Fifth, some DTAs were shorter than others because of subject control being gained quickly but were compared despite this time difference. In soccer, for example, quantity of HAEs recorded in actual versus scheduled play time were significantly higher [6]. This is an unavoidable limitation, as the assessment ends upon subject control. Assessment duration was not recorded but can be in future studies to explore if time has a significant impact on HAEs. Finally, instructors attempted to size- and ability-match participants during training activities, which may limit ecological validity in real-life scenarios.

This study is the first of its kind to quantify HAEs sustained by civilian LECs during the training academy. Although this training is necessary for job duties, monitoring HAEs may provide ways to improve safety during training. For example, boxing training resulted in the highest number and magnitude HAEs. Nonetheless, boxing training was conducted over the shortest period. Therefore, findings suggest that LECs may benefit from restructuring their boxing training to allow for more time to recover between training sessions. Additionally, the high magnitude HAEs suggest that LECs may benefit from having a medical provider on-site for injury assessments. Finally, the variability in number and magnitude of HAEs across LECs suggests future research should explore factors contributing to higher HAEs. Ultimately, the findings of this study highlight the importance of continuing to examine HAEs during tactical athlete training.

## Data Availability

Datasets generated and analyzed during the current study will be available in the FITBIR repository (https://fitbir.nih.gov/). Data are also available in Supplementary Material.
